# Alteration of Lipid Parameters in Patients With Subclinical Hypothyroidism

**DOI:** 10.5812/ijem.17496

**Published:** 2014-07-01

**Authors:** Bashir Ahmad Laway, Fayaz Ahmad War, Sonaullah Shah, Raiz Ahmad Misgar, Suman Kumar Kotwal

**Affiliations:** 1 Department of Endocrinology, Sher-I-Kashmir Institute of Medical Sciences, Soura, Srinagar, Kashmir, India; 2 Department of Internal Medicine, Sher-I-Kashmir Institute of Medical Sciences, Soura, Srinagar, Kashmir, India

**Keywords:** Hypothyroidism, Lipids, Hypothyroidism

## Abstract

**Background:**

Overt hypothyroidism is associated with abnormalities of lipid metabolism, but conflicting results regarding the degree of lipid changes in subclinical hypothyroidism (SCH) exist.

**Objectives:**

The aim of this study was to assess differences in lipid profile parameters between subjects with and without SCH in a north Indian population.

**Patients and Methods:**

Serum lipid parameters of 70 patients with subclinical hypothyroidism and 100 age and sex matched euthyroid controls were evaluated in a cross-sectional study.

**Results:**

Mean serum total cholesterol (TC), triglycerides (TG) and very low-density cholesterol (VLDL) were significantly higher in patients with SCH than controls (P < 0.05). Mean TC, TG and low-density cholesterol (LDL) concentrations were higher in patients with serum thyroid stimulating hormone (TSH) greater than 10 mU/L than those with serum TSH equal to or less than 10 mU/L, but this difference was not statistically significant. No association was found between serum high-density cholesterol (HDL-C) concentration and serum TSH level.

**Conclusions:**

High TC, TG and VLDL were observed in our patients with SCH.

## 1. Background

Subclinical hypothyroidism is defined as an elevated TSH concentration in presence of normal serum free thyroxine (FT4) and free triiodothyronine (FT3). It is a common disorder with a world-wide prevalence of about 7.5% to 8.5% in women and 2.8% to 4.4% in men (1). Thyroid hormones have significant effects on the synthesis, mobilization and metabolism of lipids (2). Overt hypothyroidism is associated with abnormalities of lipid metabolism, which may predispose the development of cardiovascular disorders (3). Lipid abnormalities in patients with subclinical hypothyroidism are not consistent (4). Many studies have reported significant increase in TC, and LDL-C and TG in patients with SCH (5-7). In patients with SCH, HDL-C has been reported as either low or comparable with control groups. The extent of lipid alteration in SCH has been a debate and so has been the effect of L-thyroxine substitution on these abnormalities (8, 9).

## 2. Objectives

Alterations of lipid parameters in patients with subclinical hypothyroidism are not consistent. Therefore, the aim of this study was to assess SCH association with abnormal lipid parameters in a north Indian population.

## 3. Patients and Methods

This case control study was conducted in a tertiary care hospital in North India. Seventy patients with SCH were compared with 100 age, sex and BMI matched healthy controls. Inclusion criteria were elevated TSH greater than 6.5 mU/L, normal FT4 (0.89-1.76 ng/dL) and normal free T3 (2.30-4.20 pg/mL) levels. Age, gender and BMI matched healthy people with normal thyroid functions were recruited as controls. Exclusion criteria were obese people with body mass index (BMI) greater than 30 Kg/m^2^, current smokers and alcoholics, diabetes mellitus, renal insufficiency (serum creatinine > 1.5 mg/dL) hepatic failure, diagnosed cases of hypothyroidism or those already on treatment, polyglandular disorders, thyroid cancer, people with a history of antipsychotic treatment or estrogen intake. A detailed history was obtained from all patients and controls. After an informed consent, all patients and controls were subjected to complete physical examination and following investigations: complete blood count (CBC), liver function test (LFT), kidney function test (KFT), fasting blood glucose, lipid profile like TC, TG, HDL-C, VLDL and low density lipoprotein cholesterol (LDL-C), and thyroid function tests including serum TSH, free T4 and free T3. Samples for all these investigations were taken 12 hours after an overnight fasting. CBC was evaluated with Sysmex XT-2000-I analyzer. KFT, LFT and fasting blood glucose were measured with Automatic Hitachi-912 Analyzer. To estimate lipid parameters and thyroid hormones, serum was separated from the blood by centrifugation at 3000 rpm, and stored at -70°C until analysis. TG and TC in serum samples were measured by enzymatic calorimetric method (Hitachi-912, Boehringer Mannheim System, and Germany). Calibration was performed using quality control sera. HDL-C in sera was measured by phosphotungstic acid and magnesium chloride method. LDL-C, VLDL and chylomicrons were precipitated from sera using phosphotungstic acid and MgCl_2_, leaving HDL-C in the supernatant. HDL the supernatant was estimated as TC. The quality control was established using Precilip EL and precinorm. LDL-C and VLDL were measured by using the Friedewald’s formula (10). Serum TSH was measured by immuno-radiometric assay (IRMA). Free T4 and free T3 were measured by chemiluminescence method (11). Hormonal assays were performed using commercial kits in duplicate and according to supplier protocols (Shinjin Ind Co. Ltd. Inchon, Korea for TSH; and Dia Sorin Stillwater MN for FT3 and FT4).

### 3.1. Statistical Analysis

All the continuous variables were expressed as mean ± SD.. In addition, categorical variables were analyzed by chi-square test. All the results were discussed at 5% level of significance; P value < 0.05 was considered significant. Statistical Package for Social Sciences version 21.0 (SPSS Inc., Chicago, IL, USA,) was used for statistical analysis.

## 4. Results

The mean age and BMI of patients with SCH and healthy controls were comparable (The mean age of patients was 36.5 ± 10.1 years, while the mean age of controls was 34.5 ± 10.3 years (P value = 0.208). BMI of patients was 23.67 ± 2.39 kg/m^2^ and BMI of controls was 23.06 ± 2.53 kg/m^2 ^(P value = 0.066). There was a female preponderance both in patients with SCH and controls. Mean serum TC, TG and VLDL in subjects were significantly higher than controls (Table 1). Mean serum TC in subjects was 182.91 ± 41.01 mg/dL versus 170.19 ± 34.36 mg/dL in controls (P value = 0.03). Mean serum TG in subjects were 173.79 ± 99 mg/dL versus 138.67 ± 57.40 mg/dL in controls (P value = 0.00). Mean serum VLDL in subjects was 34.83 ± 19.75 mg/dL versus 28.12 ± 11.36 mg/dL in controls (P value = 0.00). There was no significant difference in LDL and HDL-Cholesterol between patients with SCH and controls (Mean serum TC, TG and LDL-C concentrations were higher in patients with serum TSH greater than 10 mU/L than those with serum TSH equal to or less than 10 mU/L, but this difference was not statistically significant (Table 2). Mean serum TC in patients with TSH greater than 10 mU/L was 192.50 ± 35.04 mg/dL versus 173.86 ± 44.55 mg/dL in patients with serum TSH equal to or less than 10 mU/L (P value = 0.055). Mean serum TG in patients with TSH greater than 10 mU/L was 187.21 ± 115.33 mg/dL versus 161.14 ± 80.255 mg/dL in patients with serum TSH equal to or less than 10 mU/L (P value = 0.274). Mean serum LDL-C in subjects with TSH greater than 10 mU/L was 112.66 ± 35.52 mg/dL versus 98.64 ± 39.61 mg/dL in subjects with serum TSH equal to or less than 10 mU/L (P value = 0.123). However, no association was found between serum HDL-C concentration and serum TSH level. VLDL was slightly elevated in patients with serum TSH > 10 mU/L than those with serum TSH ≤ 10 mU/L (Table 2).

**Table 1.** Baseline Characteristics and Serum Lipid Profile in Patients with Subclinical Hypothyroidism and Healthy Controls^a^

Parameter	Patients With SCH, n = 70	Healthy Controls, n = 100	P Value
**Age, y**	36.5 ± 10.1	34.5 ± 10.3	0.208
**BMI, Kg/m** ^**2**^	23.67 ± 2.39	23.06 ± 2.53	0.066
**Total Cholesterol, mg/dL**	182.91 ± 41.01	170.19 ± 34.36	0.03
**Triglyceride, mg/dL**	173.79 ± 99.00	138.67 ± 57.40	0.00
**LDL-Cholesterol, mg/dL**	105.45 ± 38.07	99.52 ± 31.70	0.27
**HDL-Cholesterol, mg/dL**	42.27 ± 7.77	41.89 ± 7.52	0.75
**VLDL, mg/dL**	34.83 ± 19.75	28.12 ± 11.36	0.00

^a^ Abbreviations: BMI, body mass index; HDL, high density lipoprotein; LDL, low density cholesterol; SCH, subclinical hypothyroidism; VLDL, very low-density lipoprotein

**Table 2.** Lipid Parameters Based on Serum TSH Level in Patients With Subclinical Hypothyroidism^a^

Lipid Fraction, mg/dL	TSH (> 6.5 to ≤ 10), mU/L	TSH (> 10), mU/L	P Value
**Total cholesterol**	173.86 ± 44.55	192.50 ± 35.04	0.055
**Triglyceride**	161.14 ± 80.25	187.21 ± 115.33	0.274
**LDL-cholesterol**	98.64 ± 39.61	112.66 ± 35.52	0.123
**HDL-cholesterol**	42.03 ± 7.37	42.53 ± 8.328	0.790
**VLDL-cholesterol**	32.36 ± 15.97	37.45 ± 23.06	0.284

^a^ Abbreviations: HDL, high density lipoprotein; LDL, low density cholesterol; TSH, thyroid stimulating hormone; VLDL, very low density lipoprotein

## 5. Discussion

Overt hypothyroidism is associated with increased risk of cardiovascular disease, which is attributed to increased TC and LDL-C. Elevation of plasma LDL-C is due to impaired clearance of LDL, probably reflecting decreased LDL receptor expression (12). Multiple studies over the last 20 years have focused on associations between SCH and serum lipids, which has remained incompletely understood. Inconsistent results have been reported in literature regarding the association between SCH, serum lipids and cardiovascular disease (13, 14). Among 8586 adults from the National Health and Nutrition Examination Survey III database, SCH was not associated with alterations in TC, LDL-C, TG, or HDL-C after adjustment for age, race, sex, and using lipid-lowering drugs (15). Vierhapper et al. reported that there were no significant differences in serum TC, LDL-C, HDL-C, or TG between patients with SCH and the euthyroid control group (16). By contrast, a number of studies showed that TC, LDL-C, and TG were elevated in SCH compared with controls. In a population-based sample of 2799 elderly subjects, SCH was associated with elevation in total cholesterol (17). Among 25862 participants in a statewide health fair in Colorado, fasting TC, TG, and LDL-C levels were significantly greater in patients with SCH than those euthyroid subjects (1). In a community-based study of 2108 participants, serum TSH was positively correlated with TC, TG, and LDL-C, but these associations were no longer observed after adjustment for age and sex. In this study, no associations were observed between serum TSH and HDL-C (6). Among 1534 Chinese adults, patients with SCH had significantly higher TG and lower HDL-C than euthyroid individuals (18). Our cohort of people with SCH had significantly higher levels of TC and TG as compared to control subjects. In the present study, significant difference in mean values of LDL-C and between patients and controls was not found, which was observed in other studies (18). Exclusion of current smokers from our study, may partly explain lack of significant difference in LDL-C between patients and controls. Smoking is a potential modifier of the association between thyroid status and serum lipids; serum LDL-C and TC are approximately 25% higher in hypothyroid smokers compared to hypothyroid nonsmokers (19). HDL-C has been variably reported to be low or unchanged in SCH (6, 18). We found no effect of SCH on mean HDL-C. Lipid pattern is more abnormal in SCH individuals with serum TSH greater than 10 mU/L (20, 21). In the present study, total TC, TG and LDL-C, concentrations were higher in patients with serum TSH greater than 10 mU/L than those with serum TSH equal to or less than 10 mU/L. In one study from our institute, serum levels of TC and TG were significantly higher in women with polycystic ovary syndrome and SCH compared to women with polycystic ovary syndrome and normal thyroid functions (22). Marked changes in lipid profile were observed in patients with autoimmune diseases (23). Studies have shown association between increase in TC, TG, lipoproteins and thyroid autoimmunity, while one study showed no association between these two (24, 25). We did not evaluate the presence of thyroid auto-antibodies in our patients. Abnormalities in lipid parameters demonstrated in the present study could be related to some genetic alterations inherent in the population.

There can be multiple possible reasons for disparate results of studies. These include differences in patients’ ages, ethnicity, gender, and the degree and duration of hypothyroidism across studies. In addition, most observational studies did not adjust the differences in insulin resistance and smoking behavior, which were identified as potential modifiers of the association between thyroid status and serum lipids. LDL-C elevation in hypothyroid patients is enhanced in smokers and patients with insulin resistance (19, 26). High TC, TG and VLDL were observed in our patients with SCH. There are conflicting results about lipid profile pattern and SCH. This might be due to difference in population studied as well as differences in age, gender and ethnicity. Because of cross sectional nature of the present study, it is difficult to ascribe causality to any association we have found. Further evaluation of this association with longitudinal data would be necessary to support a causal link.
